# Determinants of glaucoma awareness and knowledge in urban Chennai

**DOI:** 10.4103/0301-4738.55073

**Published:** 2009

**Authors:** Ramesh Ve Sathyamangalam, Pradeep G Paul, Ronnie George, Mani Baskaran, Arvind Hemamalini, Raj V Madan, J Augustian, Raju Prema, Vijaya Lingam

**Affiliations:** Glaucoma Project, Vision Research Foundation, Sankara Nethralaya, Chennai, India

**Keywords:** Awareness, glaucoma, knowledge, India, population-based study

## Abstract

**Aim:**

To assess the awareness and knowledge levels about glaucoma and its determinants in an urban population of Chennai in south India.

**Materials and Methods:**

Chennai glaucoma study (CGS) was a population based prevalence study to estimate the prevalence of glaucoma in a rural and urban south Indian population. A total of 3850 subjects aged 40 years or above participated in the urban arm of CGS. A systematic random sample of 1926 (50.0%) subjects completed a questionnaire that assesses their awareness and knowledge level of glaucoma. Respondents “having heard of glaucoma” even before they were contacted/recruited for the study were defined as “aware” and respondents having some understanding of the eye disease were defined as “knowledgeable”.

**Results:**

Overall 13.5% were aware of glaucoma, the age-gender adjusted rate for awareness was 13.3% (95% CI: 11.57 to 15.03). Two clinicians graded knowledge on glaucoma, based on the subject's knowledge of risk factors, definitions and treatment aspects of glaucoma. Overall 8.7% had some knowledge about glaucoma. Among those who had knowledge 0.5% had good knowledge about glaucoma, 4% had fair knowledge and 4.2% had poor knowledge. We observed a very good agreement between the clinicians in grading knowledge (k =0.92). Determinants of glaucoma awareness and knowledge were higher levels of education, females, age, religion and family history of glaucoma.

**Conclusion:**

Awareness and knowledge about glaucoma was very low among the urban population of Chennai. We have found that younger subjects and men were less aware of glaucoma. Subjects with lower levels of education were less aware and knew less about glaucoma than their counterparts. The study findings stress the need for health education for effective prevention of blindness due to glaucoma.

Glaucoma is second only to cataract as the leading cause of preventable blindness in the world. It is estimated that over 65 million people throughout the world are affected by glaucoma.[1] Glaucoma causes irreversible blindness and many (50%) of the affected people are unaware of their condition.[2] We had previously reported the prevalence of primary open angle and angle closure glaucoma in the rural and urban south Indian population; in both these population over 90% of the glaucoma patients were unaware about the disease.[3,4] Increased awareness about glaucoma will increase case detection and will thereby reduce blindness due to glaucoma. Social perceptions of health have changed globally; there is an impetus to move towards good health by using resources for preventive measures. Governmental agencies and several non-governmental organizations are looking to reduce the risk factors for ocular diseases, educate the public to understand the need to improve their health status, and are teaching individuals how to increase their own ability to maintain well being.[5]

Published evidence indicates that late diagnosis of glaucoma is an important risk factor for subsequent blindness and is associated with poor knowledge about the condition.[6] The referral source is an important contributing factor for early diagnosis. Patients referred from optometrists with a diagnosis of glaucoma are more likely to be in the early stages of the disease.[7] Referral patterns in India are quite different from the West. One third of those who become blind due to glaucoma had become visually impaired even before they had sought medical attention for their eyes.[8–10] Blindness due to glaucoma can be curbed to a certain extent by educating the masses about the condition, and thereby influencing at risk individuals to participate in regular ophthalmic care.[11]

Several studies on health behavior and health belief suggest that the patient's knowledge (or lack of knowledge) concerning eye care may play a significant role in seeking timely eye care treatment.[12–14] Till date only two publications have reported the awareness status about glaucoma in India;[15,16] their figures when compared to the West reflect the poor awareness levels in Indian population. This study was aimed at understanding the awareness and knowledge about glaucoma and its determinants in a population based sample from urban cohort of the Chennai Glaucoma Study (CGS).

## Materials and Methods

The CGS is a population-based survey to estimate the prevalence of glaucoma in a rural and urban population of Chennai. The detailed methodology of the CGS has been described elsewhere.[17] Three thousand eight hundred and fifty subjects (response rate: 80.20%) of the 4800 enumerated subjects from 5 randomly chosen administrative divisions of the Chennai city population participated in the urban arm of the CGS.

The questionnaire was administered to a subset of the urban CGS participants. A systematic random sampling technique was used, i.e. every second participant (1926 subjects) starting from the first of the 3851 registered participants was assessed for their knowledge attitudes and practice (KAP) on glaucoma. Complete data was available for 3850 subjects. Demographic details and literacy level of all subjects were obtained. A brief structured open-ended questionnaire was designed to collect information about the subject's awareness and knowledge about glaucoma. (Annexure 1) Seventy-seven percent of the CGS participants (n =1480) responded to the questionnaire completely; incomplete questionnaires were not included for analysis. The questionnaire was pilot tested and had in built consistency checks. The questionnaire was translated to the vernacular language (Tamil) and back-translated to English. The questionnaire was validated against the Hong Kong study questionnaire,[18] there was good agreement between the responses (kappa: 0.92). There was good consistency in responses provided by subjects (ICC: 0.794, 95%CI: 0.77 to 0.81). Trained social workers interviewed the urban participants of CGS and recorded their responses to questions pertaining to glaucoma awareness and correct knowledge about glaucoma. The questions were administered verbatim during the interview process, so as to avoid interviewer bias. Subject's responses were recorded in the questionnaire. The questionnaire was administered prior to the history and examination procedures for glaucoma. Details on the knowledge about glaucoma were obtained only for subjects who were aware of glaucoma.

The study was conducted between June 2001 and May 2003. Written, informed consent was obtained from all subjects, and the study was performed in accordance with the tenets of the Declaration of Helsinki. The institutional review board of the Vision Research Foundation, Chennai, approved the study.

### Awareness and knowledge about glaucoma -Definition:

The response “having heard of glaucoma” even before being contacted/recruited for the study was defined as awareness and having some understanding of the eye disease was defined as knowledge.

Respondents answered questions pertaining to risk factors for glaucoma, description of symptoms and treatment aspects. Subjects were asked to describe the conditions and asked to select the most important risk factors and treatment options from the given choices. The following risk factor options were presented in the questionnaire namely obesity, increased intraocular pressure (IOP), smoking and alcohol use, steroid use, family history, cannot say and none of the above. Treatment options presented in the questionnaire were eye drops, surgery, laser, no treatment and cannot say. Knowledge was graded as good, fair and poor by two ophthalmologists independently based on the subjects collective responses to questions on the definition of glaucoma, patho-physiological risk factors and treatment aspects.

Defining knowledge levels of glaucoma: A subject was considered to have good knowledge, if he/she was able to identify the risk factors for glaucoma such as increased IOP, family history, and steroid use and was further able to meaningfully describe the condition and identify therapies for glaucoma such as eye drops, laser peripheral iridectomy, surgery. Fair knowledge was considered if at least two of the risk factors were identified and a description on at least one treatment option was correctly provided. Subjects were considered to have poor knowledge, if they were unable to identify even a single risk factor or treatment option for glaucoma.

Greater importance was given for the risk factors and description for grading knowledge. The key words that we looked for in the description were “increased eye pressure”, “loss of side vision”. Agreement was calculated for the two Ophthalmologists in grading the respondent's knowledge. The electronic form of the data was stored in a MS access database; analysis was performed using SPSS statistical software. The Chi Square test was used to look for significant variations in knowledge and awareness about glaucoma with other studied variables. The influence of age, gender, religion, ethnicity, and economic status on the subject's knowledge and awareness of glaucoma was accessed using multiple logistic regression analysis. Age-gender adjustment was done using the Chennai urban standard population from the Census of India, 2001. A *P* value less than 0.05 was considered statistically significant.

## Results

**Figure 1:**
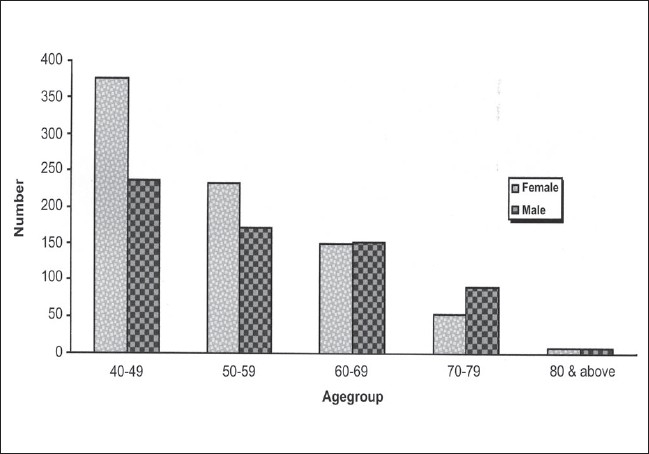
The distribution of study subjects by age group and gender (n=1480)

Out of 1926 subjects to whom the questionnaire was administered, one thousand four hundred and eighty subjects completed the questionnaire. (Response rate of 77%) Among the responders, 44% were males and 56% were females. The mean age of participants was 54 ± 11 years. Minimum age was 40 years and the maximum age was 103 years. The proportion of subjects in each age cohort decreased with increasing age for both genders; chi square *p*<0.001 [Fig. 1]. Twenty four percent of the respondents were Illiterate, 35% had primary or below primary level of education, 28% had up to secondary level education, 12% had tertiary education. Education information was not available for 1% of the respondents. Eighty three percent of the respondents were Hindus, 13% were Muslims and 4% were Christians. Mother tongue was Tamil for 82%, Telugu for 9%; Malayalam for 2%, Hindi for 3% and other languages for 4% of the respondents. A total of 200 (13.5%, 95% CI: 11.76 to 15.24) subjects were aware of glaucoma, the age gender adjusted prevalence of awareness was 13.3% (95% CI: 11.57 to 15.03) [Table 1]. Age-gender adjustment was done using Chennai Urban standard population from Census of India, 1991. Only 10.95% (95% CI: 9.61 to 12.29) of the subjects felt that glaucoma was treatable. Among these 200 subjects, 136 subjects (68%) of them had heard about glaucoma but could not describe the condition.

**Table 1:** Frequency distribution of awareness and knowledge of glaucoma among study participants

Variables	Total n = 1480 Yes (%)
**Awareness**	
Have you ever heard of the eye condition glaucoma	200 (13.5)
Not aware of glaucoma	1280 (86.5)
Is glaucoma treatable?	148 (10.0)
**Knowledge**	
***Risk factors for glaucoma:***	
One factor	
Obesity	2 (0.14)
Increased IOP	120 (8.11)
Steroids	0
Chronic smoking + alcohol using	6 (0.405)
Family history of glaucoma	21 (1.42)
Diabetes	20 (1.35)
*Two factors*	
IOP and steroids	7 (0.47)
IOP and family history	23 (1.55)
Steroids and family history	6 (0.41)
*Three factors*	
IOP, steroids and family history	4 (0.27)
Risk factors not known	31 (15.5)
Meaningful description of glaucoma	
*Key words*	
Increased IOP, eye pressure	22 (1.48)
Loss of side vision	7 (0.47)

IOP: intraocular pressure

There was no association between age group and awareness (*p*=0.108), however after adjusting for gender the odds for glaucoma awareness increase with increasing age. Subjects in the age groups 60 - 79 years were 2.7 to nearly 3 times more likely to be aware about glaucoma when compared to 40 - 49 year olds [Table 2], females when compared to males seemed to have higher levels of awareness, (Adjusted OR- 1.54; 95% CI: 1.06 to 2.25). Education levels clearly seemed to influence glaucoma awareness independent of age, gender, religion and ethnicity. (*p*<0.001) The wider confidence intervals are due to the fewer number in the literacy and illiterate categories. Subject's awareness of glaucoma was not influenced by the disease state (*p*=0.347) (either glaucoma or diabetes). Hindus were 4 times more likely to be aware of glaucoma when compared to Muslims. (Adjusted OR: 4.16, 95% CI: 1.41 to 12.5). The language spoken at home does not seem to influence the awareness about glaucoma in our sample. (*p*=0.234) People with family history of glaucoma when compared to those without family history were more likely to be aware of glaucoma (Adjusted OR:5.51, 95% CI: 2.21 to 13.75) [Table 2].

**Table 2:** Determinants of awareness and knowledge about glaucoma

Variable	Awareness (n=1480)			Knowledge (n=200)		
		
	Aware (%)-n=200	Not Aware (%): n=1280	Odds Ratio (95% CI)	Knowledge (%): n=67	Non - Knowledge (%): n=133	Odds Ratio (95% CI)
Gender						
Female	11	89	1	4	96	
Male	17	83	0.65 (0.44 to 0.95)	6	94	0.75 (0.42 to 1.33)
Age Group						
40-49	11	89	1	4	96	
50-59	14	86	1.85 (1.20 to 2.85)	4	96	0.96 (0.48 to 1.89)
60-69	17	83	2.69 (1.64 to 4.39)	6	94	1.34 (0.65 to 2.77)
70-79	15	85	3.09 (1.62 to 5.90)	5	95	1.58 (0.60 to 4.12)
80 and above	13	87	1.94 (0.27 to 13.76)	6	94	1.34 (0.11 to 16.25)
Literacy Categories						
Illiterate	0	100	1	0	100	
Primary	5	95	24.88 (3.36 to 184.17)	2	98	8.93 (1.14 to 69.86)
Secondary 203.14)	21	79	108.01 (14.83 to 786.52)	7	93	26.9 (3.55 to
Tertiary 560.26)	48	52	435.78 (58.47 to 3247.94)	16	84	71.8 (9.20 to
Glaucoma status						
Non-Glaucoma persons	13	87	1	4	96	
Glaucoma persons	16	84	1.18 (0.78 to 1.80)	7	93	1.82 (1.01 to 3.26)
Diabetic Status						
Non-Diabetic persons	13	87	1	4	96	
Diabetic persons	17	83	0.82 (0.54 to 1.25)	6	94	1.09 (0.59 to 2.00)
Religion						
Hindu	15	85		5	95	
Christian	16	84	0.79 (0.32 to 1.91)	7	93	1.08 (0.30 to 3.86)
Muslim	2	98	0.24 (0.08 to 0.71)	2	98	0.91 (0.29 to 2.81)
Mother Tongue						
Tamil	13	87	1	4	96	
Telugu	15	85	0.86 (0.49 to 1.50)	5	95	1 (0.43 to 2.34)
Malayalam	24	76	1. 40 (0.61 to 3.21)	10	90	1.63 (0.51 to 5.21)
Hindi	18	82	0.48 (0.16 to 1.47)	0	100	NA
Others	14	86	1.26 (0.52 to 3.05)	5	95	1.14 (0.31 to 4.18)
Family History of Glaucoma						
No	20	80	1	2	98	1
Yes	45	55	5.51 (2.21 to 13.75)	10	90	4.57 (1.77 to 11.78)

**Age and gender adjusted Odds ratio (OR), *95% CI: Confidence Interval for the Odds ratio. NA: OR could not be calculated as none of the Hindi speaking subject had significant knowledge.

Knowledge about the risk factors for glaucoma among the study participants is presented in Table 1. Among the study participants only 8% considered increased IOP as a risk factor. Of the 200 people who were aware of glaucoma, 64.5% had knowledge about glaucoma.

In our effort to find out the predictors of glaucoma knowledge, we categorized subjects with good and fair knowledge as “subjects with knowledge” and those with poor knowledge as “subjects without knowledge” about glaucoma. Of the entire population, 8.7% had knowledge about glaucoma. Among them 0.5% had good knowledge about glaucoma, 4% had fair knowledge and 4.2% had poor knowledge. We observed a very good agreement between the clinicians in grading knowledge (k =0.92). Table 3 shows the subjects knowledge level on treatment options for glaucoma. Knowledge levels about glaucoma were similar across both the males and females. (adjusted OR for males: 1.33, 95% CI: 0.75 to 2.38) [Table 2]. Age groups were not associated with knowledge of glaucoma (*p*=0.771).

**Table 3:** Frequency distribution of treatment procedure for glaucoma among study participants

Variables therapies for treating glaucoma	Total n = 1480 Yes (%)
Aware of only a single therapy	
Eye drops	66 (4.5)
Surgery	21 (1.4)
Laser treatment	34 (2.3)
Aware of two therapies	
Eye drops and Surgery	6 (0.4)
Eye drops and Laser	10 (0.7)
Surgery and Laser	10 (0.7)
Aware of more than two therapies	
Eye drops, Surgery and Laser	15 (1.0)
Treatment not known	32 (2.16)
Can't say / No Answer	6 (0.4)

We analyzed the effect of education on the subject's knowledge levels about glaucoma. Subjects without any formal education were considered as “illiterates” and the other categories are those with primary (1-5 years of education), secondary (5–10 years of education) and tertiary levels of education (education levels from 10+2 to degree or more). Subjects with primary level education were 9 times more knowledgeable about glaucoma than illiterates. (adjusted OR:8.93; 95% CI: 1.14 to 69.86), people with secondary education were 27 times more likely to be knowledgeable than illiterate subjects and subjects with tertiary levels of education were 72 times more likely to be knowledgeable than the illiterates. (Adjusted OR: 71.8; 95% CI: 9.2 to 560.26) [Table 2].

Glaucoma patients were more likely to be knowledgeable than the normal subjects. (Adjusted OR: 1.82, 95% CI: 1.01 to 3.26) [Table 2]; however diabetic status did not significantly predict knowledge about glaucoma. (Adjusted OR: 1.09, 95% CI: 0.59 to 2.00). Likewise religion and mother tongue did not influence the knowledge about glaucoma. (*p*=0.655) [Table 3]. Persons with family history of glaucoma were 5 times more likely to be knowledgeable about glaucoma when compared to those without family history of glaucoma. (Adjusted OR: 4.57, 95% CI: 1.77 to 11.78) [Table 2]. Major determinants of glaucoma awareness were higher levels of education, age and Hindus and that of knowledge of glaucoma were higher levels of education and glaucoma patients.

## Discussion

Glaucoma is an irreversible and asymptomatic condition until the advanced stage. Early detection and treatment plays a pivotal role in control of blindness due to glaucoma. One third of the patients who had become blind from glaucoma had done so even before they had sought medical attention.[8] Awareness of eye diseases in a urban Indian population[15] and awareness of glaucoma in a rural Indian population have been previously reported.[16] Awareness does not mean that subject knows everything about the disease; it just means that he/she has heard about the condition.

Previous studies have showed that even though most people claim to be aware of the condition less than a percent could describe its symptoms or patho-physiology correctly.[18] Knowledge about the disease would be more useful, as it is presumed to influence their ocular health-seeking pattern. Age and sex adjusted prevalence of primary glaucoma in urban Chennai was 4.39%.[2,3] The age and sex adjusted glaucoma awareness rate among the general population of Chennai was 13.3% and only 8.7% of the Chennai residents had some knowledge about glaucoma.

India has divisions on many levels, a few of these being religion, language and inherent attitudes to health. The demographics and lifestyles differ from state to state and from rural to urban to tribal ways of life.[19] Urban Chennai residents (13.3%) seem to be more aware about glaucoma when compared to their counterparts in Hyderabad (2.4%).[15] The observed difference in glaucoma awareness could be explained by the different definitions used (Andhra Pradesh Eye Disease Study - age > 15 years) across the two studies, the difference in study methodology and tools, and also by the diversity of Indian culture. Knowledge level about glaucoma between the Hong Kong (10.2%)[18] and Chennai (8.7%) populations were comparable. Dissimilarities exist in awareness levels between the two countries; we presume it could be largely due to easy access to health care and better utilization of eye care services for glaucoma in Hong Kong [Table 4]. In our study, glaucoma awareness was higher than in Hyderabad but lower than reports from the developed Nations (United States, Australia, Singapore and Hong Kong).[6] In well-educated western population (Blue Mountains Eye Study population), 93% of participants were aware of glaucoma [Table 4], the proportion of undiagnosed glaucoma among all cases was found to be very high;[20] these undiagnosed glaucoma cases are a cause for alarm considering the low levels of awareness regarding the disease in our population.

**Table 4:** Glaucoma awareness and knowledge levels across the globe

Author	Year	Country	Study population	Awareness of glaucoma %	Knowledge of glaucoma %
Present study	2004	India	Urban population - Adults above 40 years	13.30	8.70
Dandona *et al*[15]	2001	India	Urban population - Above 15 yrs	2.30	Not Reported
Krishnaiah *et al*	2005	India	Rural population: Above 15 yrs	0.27	0.012
Gasch *et al*[21]	2000	United States	General eye service patients - All Ages	72	Not Reported
Mitchell *et al*[20]	1996	Australia	Community - Adults above 49 yrs	93	Not Reported
Livingston *et al*[22]	1995	Australia	Community - Adults above 40 yrs	70	Not Reported
Michielutte[14]	1984	United States	Community - Above 14 yrs	81	Not Reported
Saw *et al*[6]	2003	Singapore	Tertiary eye hospital patients - Adults 35 yrs and above	23	Not Reported
Lau *et al*[18]	2002	Hong Kong	Community - Adults above 40 years	78.40	10.20

In our study, we observed that people with family history of glaucoma and women were more likely to be aware and had good knowledge of glaucoma. Illiterates and people with below primary level of education were more likely to be unaware about glaucoma; this was consistent with studies done elsewhere.[6,14,15,18,21,22] and indicates the lack of education about glaucoma among those who are at risk. It calls for urgent health education on glaucoma, targeting initially, the population at risk.

Health promotion and communicating risk is a key public health strategy.[5,11,23] Public awareness of vision care especially glaucoma is very low. Effective health education about eye care may influence the behavior of individuals, to consider regular ocular care. Communicating visual prognosis by primary health clinicians and primary eye care practitioners would help enhance the knowledge and compliance among glaucoma patients. The education programs need to target the known cases, due to their disease status or other epidemiological risk characteristics such as people with family history of glaucoma, aged people and angle closure. The aims of education should focus not only on modifying individual's perception of risk of vision loss, but also on providing information regarding the benefits of early detection and treatment. In addition, education programs should also be oriented towards the involvement of friends and family members in supporting the seeking of eye care and in alleviating the fear or anxiety concerning treatment.[5] It is important to note that the benefits of eliminating barriers to access can be fully realized only when the issue of adequate utilization of preventive services is also addressed. Studies across the globe have clearly documented the potential cost savings associated with regular preventive eye care as compared to cost of vision loss.[11] Community level programs and initiative taken as part of the World Glaucoma Day in increasing awareness on glaucoma through various media and setting up patient awareness groups would help improve the awareness in this population.[24]

It is also essential to ensure early detection through ‘opportunistic case detection’ by performing a comprehensive eye examination at every available instance, and commencing treatment or appropriate referral so as to meet the increased demand for services that is expected following effective health promotion and raised awareness about glaucoma.

In summary, awareness levels and knowledge about glaucoma were very low in our population. Younger subjects and men were less aware of glaucoma. Subjects with lower levels of education were less aware and knew less about glaucoma than their counterparts. The study findings stress the need for health education to effectively prevent blindness due to glaucoma.

## Annexure 1: Questionnaire

Awareness about Glaucoma (even before being contacted/recruited for the study) Have you ever heard of the eye condition glaucoma?

  Yes

  No

  Can't say

Is Glaucoma treatable?

  Yes

  No

  Can't say

Knowledge about Glaucoma

Tick the possible risk factors for Glaucoma.

Obesity

Increased Intra ocular pressure

Steroids

Chronic Smoking and Alcohol intake

Family history of glaucoma

Diabetes.

None of the above

Can't say

Description of Glaucoma

How would you describe it? (Record all mentioned- Symptoms)

What are the therapies for treating Glaucoma that are currently available? Tick the appropriate choices.

Medicines- Eye drops

Surgery

Laser treatment

No treatment available.

Can't say.
